# IRF2 loss is associated with reduced MHC I pathway transcripts in subsets of most human cancers and causes resistance to checkpoint immunotherapy in human and mouse melanomas

**DOI:** 10.1186/s13046-024-03187-5

**Published:** 2024-10-02

**Authors:** G. Sari, K. Dhatchinamoorthy, L. Orellano-Ariza, L. M. Ferreira, M. A. Brehm, K. Rock

**Affiliations:** 1 Department of Pathology, UMass Chan Medical School, 55 Lake Avenue North, Worcester, MA 01655 USA; 2 Program in Molecular Medicine, Diabetes Center of Excellence, UMass Chan Medical School, Worcester, MA USA

**Keywords:** Antigen presentation, MHC I, IRF2, Immunotherapy, Melanoma

## Abstract

**Background:**

In order for cancers to progress, they must evade elimination by CD8 T cells or other immune mechanisms. CD8 T cells recognize and kill tumor cells that display immunogenic tumor peptides bound to MHC I molecules. One of the ways that cancers can escape such killing is by reducing expression of MHC I molecules, and loss of MHC I is frequently observed in tumors. There are multiple different mechanisms that can underly the loss of MHC I complexes on tumor and it is currently unclear whether there are particular mechanisms that occur frequently and, if so, in what types of cancers. Also of importance to know is whether the loss of MHC I is reversible and how such loss and/or its restoration would impact responses to immunotherapy. Here, we investigate these issues for loss of IRF1 and IRF2, which are transcription factors that drive expression of MHC I pathway genes and some killing mechanisms.

**Methods:**

Bioinformatics analyses of IRF2 and IRF2-dependent gene transcripts were performed for all human cancers in the TCGA RNAseq database. IRF2 protein-DNA-binding was analyzed in ChIPseq databases. CRISRPcas9 was used to knock out IRF1 and IRF2 genes in human and mouse melanoma cells and the resulting phenotypes were analyzed in vitro and in vivo.

**Results:**

Transcriptomic analysis revealed that IRF2 expression was reduced in a substantial subset of cases in almost all types of human cancers. When this occurred there was a corresponding reduction in the expression of IRF2-regulated genes that were needed for CD8 T cell recognition. To test cause and effect for these IRF2 correlations and the consequences of IRF2 loss, we gene-edited IRF2 in a patient-derived melanoma and a mouse melanoma. The IRF2 gene-edited melanomas had reduced expression of transcripts for genes in the MHC I pathway and decreased levels of MHC I complexes on the cell surface. Levels of Caspase 7, an IRF2 target gene involved in CD8 T cell killing of tumors, were also reduced. This loss of IRF2 caused both human and mouse melanomas to become resistant to immunotherapy with a checkpoint inhibitor. Importantly, these effects were reversible. Stimulation of the IRF2-deficient melanomas with interferon induced the expression of a functionally homologous transcription factor, IRF1, which then restored the MHC I pathway and responsiveness to CPI.

**Conclusions:**

Our study shows that a subset of cases within most types of cancers downregulates IRF2 and that this can allow cancers to escape immune control. This can cause resistance to checkpoint blockade immunotherapy and is reversible with currently available biologics.

**Supplementary Information:**

The online version contains supplementary material available at 10.1186/s13046-024-03187-5.

## Introduction

Classical studies of tumorigenesis in mice found that the incidence of cancers was markedly increased in mice that lacked the adaptive immune system [1–3]. Moreover, when these tumors were transplanted into wild-type (WT) mice, most were rejected, indicating that these cancers were still immunogenic and could be eliminated by the adaptive immune system. In contrast, most tumors that arose in mice with an intact adaptive immune system were ones that would grow when transplanted into WT mice and therefore had evolved to evade immune rejection [1–3]. Therefore, to progress most tumors had to evade adaptive immune responses.

CD8 T lymphocytes are the major adaptive immune cells that kill tumors. They identify cancers by detecting the presence of peptides from tumor antigens that are displayed on the cell surface bound to MHC I molecules [4]. These immunogenic peptides are generated upon degradation of their source proteins and loaded onto MHC class I in a complex set of mechanisms [4] in by proteasomes and immunoproteasomes (proteasomes containing ß1i, ß2i & ß5i subunits). The resulting peptides are transported into the lumen of the endoplasmic reticulum (ER) by a dedicated peptide transporter (TAP) wherein they may be further trimmed by ER aminopeptidases (ERAP1 & ERAP2). In parallel the MHC I complex (MHC I heavy chain + ß2-microglobulin) assemble in the ER and localize into a peptide-loading complex that contains a chaperone (calreticulin), an oxidoreductase (ERP57), and a protein (tapasin) that helps load high affinity peptides onto the MHC I molecules; another such peptide-loading/editor molecule, TAPBPR is present outside of the peptide-loading complex [4]

While there are many mechanisms by which tumors evolve to evade CD8 T cells (e.g., expressing inhibitory molecules like PDL-1 (CD274) or becoming resistant to killing), one very common mechanism is the loss of expression of MHC I molecules [4, 5]. This latter mechanism makes cancers harder to be detected and killed by CD8 T cells [5]. The loss of MHC I is facilitated by the fact that the entire MHC I pathway is not essential for tumor growth or survival and therefore is dispensable. Moreover, loss of almost any of the MHC I peptide-loading pathway components described above results in a reduction of MHC I levels. Indeed, MHC I^low^ cancers can have a variety of lesions in the MHC I pathway [4, 5]. Thus, an important question is which lesions that lead to low MHC I expression are associated with cancers and if they are reversible would that potentially be therapeutic [4, 5].

We initiated this study to answer the above questions for the loss of the MHC I pathway component, IRF2. IRF2 is a transcription factor was initially identified as a transcriptional repressor. However, in a previous forward genetic screen, we found that IRF2 was also a transcriptional activator of multiple MHC I pathway genes, at least in mouse dendritic cells and some cancer cell lines. We found IRF2 was also an activator of gene expression for caspase 7 (RNAseq data in ref. [6], but not pointed out) and gasdermin D [6]. Caspase 7 is an effector caspase (capable of killing cells) that is activated when human CD8 T cells or NK cells deliver granulysin into their target cells [7], and gasdermin D is a pore protein involved in pyroptotic cell death [8]. In contrast, IRF2 functions as a repressor of some genes, most notably CD274, the ligand of the checkpoint receptor PD1, at least in some cells [6, 9, 10]. Consequently, loss of IRF2 could render cancers both harder to see (loss of MHC I) and harder to kill (loss of caspase 7 and increased PDL-1).

## Methods

### Cells

B16F0 (ATCC-CRL-6322) cell line was grown in Dulbecco's Modified Eagle's Medium (DMEM, Gibco) supplemented with 10% FBS (Hyclone), 1% NEAA (Gibco), 1% HEPES (Gibco), 1% Antibiotic-Antimycotic (Gibco), and 50 nM 2-ME (Sigma). For CRISPR-Cas9 targeting, cells were passed into 6 well plates (5-6x10^5^ cells/well) and cultured overnight before spin infection. Antibiotic selection for CRISPR-Cas9 knockout cells was done for seven days in media containing 10 μg/mL blasticidin (Invivogen) or 7.5 μg/mL puromycin (Invivogen) and cells were grown in a 10% CO2 atmosphere at 37°C. For IFNα treatment, B16F0 cells were treated with 1-100 ng/mL mouse IFN-α (Bioligands) in culture media for 5-72h.

A patient melanoma tumor was obtained from the UMass Chan Medical School Cancer Avatar Institute (IRB ID: H00004721) and patient-derived xenograft (PDX) line was established by passage in NSG mice (AV17). For tumor implants the PDX melanoma was processed into 2 × 2-mm^3^ pieces or a single-cell suspension, and either a tumor fragment or cells (2.5 × 10^6^) were transplanted subcutaneously to the right flank of humanized NSG [11]. The mice were monitored for tumor growth using a caliper.

To generate CRISPR-Cas9 knockouts, AV17 tumors that were grown in NSG mice were cut into small pieces, incubated in RPMI 1640 containing 30 μg/ml Liberase TM (Roche) and 20 μg/ml DNAse type I (Sigma) for 45 min, and passed through a 100 μm cell strainer. After centrifugation, cells were re-suspended in culture medium (RPMI 1640, 10% FCS, 1% NEAA (Gibco), 1% HEPES (Gibco), 1% Antibiotic-Antimycotic (Gibco), 50 nM 2-ME (Sigma), 10 ng/mL recombinant human IGF-I and EGF (Peprotech) and cultured for additional 3-4 days before being targeted with CRISPR-Cas9 vectors. Antibiotic selection for CRISPR-Cas9 knockout cells was done for three days in media containing 5 μg/mL blasticidin (Invivogen) and cells were grown in a 10% CO_2_ atmosphere at 37°C.

### Mice

All mouse strains were maintained in specific pathogen-free facilities at the UMass Chan Medical School in accordance with approved guidelines set forth by the UMass Chan Department of Animal Medicine and Institutional Animal Care and Use Committee (protocol number: 201900341). C57BL/6 and NOD.*Cg-Prkdc*^*scid*^*Il2rg*^*tm1Wjl*^*/SzJ* (NOD-*scid IL2rγ*^*null*^ (NSG) mice were acquired from Jackson Laboratories.

Human umbilical cord blood (UCB) was obtained in accordance with the Committee for the Protection of Human Subjects in Research guidelines of the University of Massachusetts Chan Medical School. UCB was provided by the medical staff of the University of Massachusetts Memorial Umbilical Cord Blood Donation Program. Four‐week‐old female NSG mice were irradiated with 100 cGy, and irradiated mice were injected IV with CD3‐depleted human UCB containing 5 × 10^4^ CD34^+^ HSC [12].At the indicated time points, flow cytometry analyses of the blood of HSC recipients quantified the engraftment of the human immune system. For experimental studies, mice with >20% peripheral human CD45+ cells and >10% human CD3+ T cells were used.

### Tumor transplant experiments

Female, 4-6 weeks old C57BL/6 mice were injected with 0.5x10^6^ WT or knockout B16F0 cells subcutaneously on the right flank. Tumor growth and mouse condition were checked three times a week. For the treatments, mice were injected with 200 μg/mouse/injection InvivoPlus anti-mouse PD-1 antibody (29F.1A12, BioXcell), InvivoPlus rat IgG2a isotype control (2A3, BioXcell) and high molecular weight Poly(I:C) (InvivoGen) twice a week, intraperitoneally.

For the tumor implants of the PDX melanomas, WT (10^7^ cells) or IRF2KO (1.25 × 10^6^ cells) were transplanted subcutaneously to the right flank of humanized NSG [11]; lower numbers of IRF2KO cells were used because of faster growth. The mice were monitored for tumor growth using a caliper. For the treatments, mice were injected with 1mg/mouse/injection Keytruda (Pembroluzimab, Merck & Co., Inc.) once a week, intravenously.

In survival experiments, the end point was when mice were moribund or when tumors ulcerated or grew ≥ 1500 mm^3^ or ≥1.5 cm in one dimension.

### Cell surface staining using flow cytometry analysis

Cells were stained with Zombie Violet fixable viability kit (Biolegend) to gate out the dead cells. Where indicated, mouse cells were blocked with 2.4G2 and stained for surface MHC class I levels with two-step staining with Y3 hybridoma supernatant followed by 1:500 donkey-anti-mouse Alexa 647 antibody (Life Technologies), for surface PD-L1 levels with anti-PD-L1-PE antibody (BioLegend, 10F.9G2), or with isotype controls (eBioscience mouse IgG2a-APC eBM2a, eBioscience rat IgG2b κ-PE eB149) at 1:200 dilutions. B16F0 parental cells were sorted on BD LSRII to isolate high MHC class I expressing cells after two step staining with Y3 hybridoma supernatant and donkey-anti-mouse Alexa 647 antibody (Life Technologies) antibody.

Where indicated, human cells were stained for surface MHC class I levels with two-step staining W6/32 hybridoma supernatant followed by 1:500 donkey-anti-mouse Alexa 647 antibody (Life Technologies). Human cells were stained for surface PD-L1 levels with 1:50 mouse anti-PD-L1-PE antibody (Biolegend, 29E.2A3).

When staining single cells obtained from in vivo grown tumors, tumors were cut into small pieces, incubated in RPMI 1640 containing 30 μg/ml Liberase TM (Roche) and 20 μg/ml DNAse type I (Sigma) for 45 min, and passed through a 100 μm cell strainer. After centrifugation, cells were re-suspended in PBS and red blood cells were lysed using red blood cell lysis buffer (Boston Bioproducts). Tumor infiltrating lymphocytes were gated out using mouse or human anti-CD45 antibodies (Alexa Fluor® 700 mouse anti-human CD45 antibody (BD biosciences, HI30) or rat anti-mouse CD45 antibody (BD Biosciences, 30-F11)). Geometric MFI values were shown for illustrations.

FlowJo™ Software was used for data interpretation and data visualization.

### Plasmids

The plasmids used to target mouse IRF2 and IRF1 or to target human IRF2 were constructed by inserting the following sgRNA sequences, respectively, into the LentiCRISPRv2 plasmid: Mouse IRF2: 5’-TCCGAACGACCTTCCAAGAA-3’; Mouse IRF1: 5’-CTCATCCGCATTCGAGTGAT-3’; Human IRF2: 5’-TGCATGCGGCTAGACATGGG-3’. The LentiCRISPRv2 plus blasticidin or puromycin selection plasmid was acquired from Addgene (83480) and, unmodified, is the same as the “empty vector (EV)” plasmid.

To insert these sgRNA sequences into LentiCRISPRv2, two primers were created for each sgRNA sequence: (1) a forward primer wherein CACCG preceded the corresponding sgRNA sequence; and (2) a reverse primer where the reverse complement of the corresponding sgRNA sequence was flanked by AAAC at the 5’ end and C at the 3’ end [13, 14]. Then, these 100μM primer sets were annealed and diluted 1:50. 3μg of LentiCRISPRv2 plasmid was digested for 3hrs at 55°C with BsmBI (NEB) and removal of the 2kb filler sequence was confirmed by gel electrophoresis. The larger molecular weight band was gel extracted and quick ligated with the diluted annealed guide primers according to the manufacturer’s instructions (NEB). Stable competent E. coli (NEB) were then transformed with 2μL of the ligation product according to the manufacturer’s instructions and grown overnight at 37°C on LB + Ampicillin (100μg/mL) agar plates. Plasmids were isolated (Clontech) from individual colonies and sequenced (Genewiz-Azenta) using the primer hU6-F: 5’-GAGGGCCTATTTCCCATGATT-3’ to confirm proper insertion of the sgRNA into LentiCRISPRv2. Following CRISPR-Cas09 knockout targeting, sgRNAs and target genomic DNA sequences were checked for high indel efficiencies in transduced cells by ICE CRISPR Analysis Tool (Synthego).

### RNA isolation, generation of cDNA and real-time PCR

Tumor tissue was collected in RNAlater (Qiagen) and the cell lines were processed immediately after collection. RNA was extracted using RNeasy mini kit (Qiagen) and the samples were treated with DNAse-I enzyme to prevent genomic DNA contamination (RNase-Free DNase Set, Qiagen). cDNA was generated using the RNA to cDNA Ecodry Premix (Takara) according to the manufacturer’s protocol.

Quantitative PCR were performed using the TaqMan probes as follows: mouse Tap1 - Mm00443188_m1, mouse Tap2 - Mm01277033_m1, mouse Erap1 - Mm00472842_m1, mouse Psme1- Mm00650858_g1, mouse Irf1 - Mm01288580_m1, mouse Hprt1 - Mm03024075_m1, human IRF2 - Hs01082884_m1, human TAP1 - Hs00388675_m1, human TAP2 - Hs00241060_m1, human PSME1- Hs00389209_m1, human ERAP1 - Hs00429970_m1, human HPRT1 - HS02800695_m1. Expression of target genes was normalized to the expression of HPRT1 using the formula 2^-ΔCt^.

### Western blotting

Cell lysates were prepared in RIPA buffer with protease inhibitor (Pierce), and protein concentrations were determined by BCA assay (Pierce). 30μg of denatured samples were run on 10% reducing gels (Genscript). After transfer, PVDF membranes (Millipore) were blocked with TBS-Tween 1x + 5% milk and then blotted with rabbit anti-IRF2 antibody (Abcam ab124744) or rabbit anti-IRF1 antibody (Abcam ab186384) in TBS-Tween 1x + 2% milk overnight at 4°C. The following day, membranes were washed 3x with TBS-Tween 1x, goat-anti-rabbit HRP (Millipore) was added for 1hr at RT, membranes were washed 3x, and HRP substrate (Millipore) was added. Following exposure, membranes were stripped (Millipore), blocked, and re-blotted with mouse anti-β-actin antibody (Santa Cruz sc-47778) in TBS-Tween 1x + 2% milk overnight at 4°C. The following day, membranes were prepared as above.

## Results

### Most human cancers have strong correlations between IRF2 and MHC I pathway gene expression

**Fig. 1:**
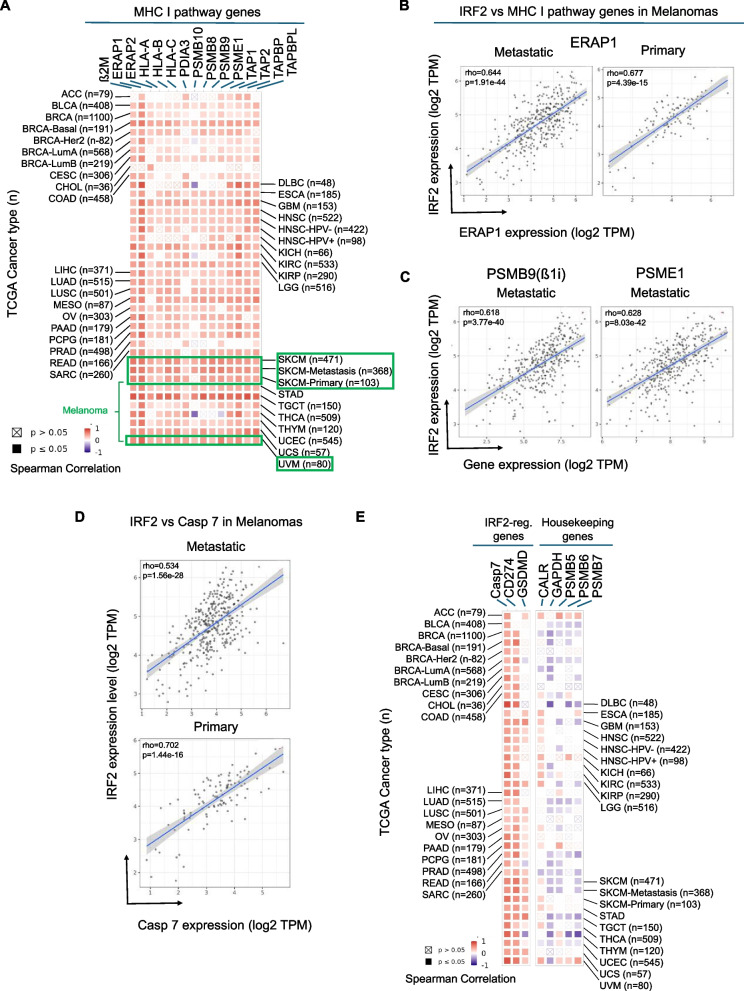
**A** Spearman correlations between transcripts of IRF2 and those of MHC I pathway genes (β2M, Erap1/2, HLA-A, HLA-B and HLA-C, PDIA3, PSMB9-10, PSME1, TAP1/2, TAPBP and TAPBPL) in tumor tissue from patients with the indicated cancer types (TCGA abbreviations). **B-D** Gene expression correlation between IRF2 vs ERAP1, PSME1, PSMB9, and Casp7 transcripts in primary and/or metastatic melanomas. **E** Spearman correlations between IRF2 transcripts and IRF2-regulated genes (Casp7, CD274 and GSDMD) and housekeeping genes (CALR, GAPDH, PSMB-7) in tumor tissue from patients with the indicated cancer types (TCGA abbreviations). **A-E** Data comes from the TCGA RNAseq database and was analyzed with TIMER [35].

A number of types of human cancers express significantly lower levels of IRF2 transcripts compared to their normal counterparts [6] and similarly almost all categories of human cancers have a subset of cases with low IRF2 levels (Supp. Fig.1). To investigate whether this variation in IRF2 expression potentially had functional consequences, we analyzed the TCGA RNAseq database to determine whether cancer cases that downregulated IRF2 transcripts levels had corresponding reductions in the expression of MHC I pathway genes (β2M, ERAP1/2, HLA-ABC, PDIA3, PSMB8-10 PSME1, TAP1/2, TAPBP and TABPL), as well as a few other IRF2-regulated genes (CASP7, CD274 and GSDMD). Remarkably, there were significant (p<0.05) positive correlations between the levels of IRF2 expression and that of virtually all of the MHC I pathway components (Fig. 1). In other words, cases with low IRF2 transcripts had correspondingly low levels of immunoproteasome subunits, TAP, ERAP1 and other pathway gene transcripts. Since this transcript data came from RNAseq databases, we didn’t have access to the primary samples to further correlate these transcript levels with corresponding protein levels. However, where it has been examined, loss of IRF2 expression from gene knock out consistently reduced MHC I protein expression and conversely IRF2 transfection increased MHC I protein expression (see below and [6]). In other words, IRF2 function may be responsible for limiting for MHC I expression.

A similar positive correlation was observed for IRF2 and Casp7 transcripts (Fig. 1D and E). In contrast, there was no positive correlation between IRF2 expression and that of the housekeeping genes GAPDH and PSMB7-9 (the active site subunits of constitutive proteasomes). Calreticulin, which plays a broad role as a chaperone for many ER proteins including MHC I, was only positively correlated with IRF2 expression in some cancer types. The correlation between IRF2 and Gasdermin D (GSMD) expression was weak or absent in a number of cancers. Different from what was seen in mouse dendritic cells [6], CD274 transcripts were not negatively correlated with IRF2 mRNA (Fig. 1E) but rather had a positive correlation in some cancers. Seeing that IRF2 expression was significantly correlated with expression of all MHC I pathway gene transcripts led us to examine whether these pathway genes had IRF1/2 binding sites. For this purpose, we examined publicly available ChIPseq data and found that all of the correlated MHC I pathway genes, with the exception of PDIA3 (ERP57), as well as CD274 and CASP7, had IRF2 and/or IRF1 bound to their 5’ region of these genes (Supp. Table-1A&B).

From these analyses, we observed that melanomas were one of the human cancers that had a subset of cases that expressed low levels of IRF2 (Supp. Fig.1) and had strong correlations between IRF2 expression and the MHC I pathway genes (Fig. 1B and C). Since melanomas are one of the cancers that can be immunogenic and a target of CD8 T cells, we further analyzed the functional consequences of IRF2 loss in these cancers.

### Loss of IRF2 reduces the expression of MHC I pathway components in human and mouse melanomas

**Fig. 2:**
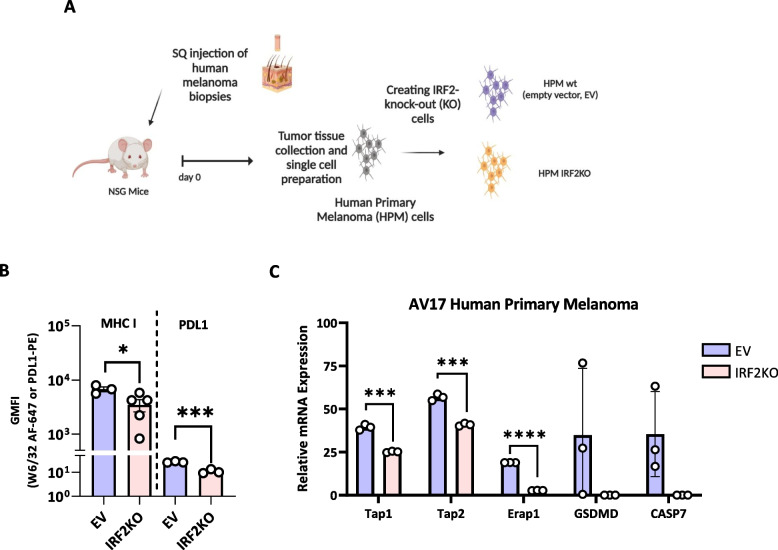
Loss of IRF2 reduces the expression of MHC I pathway components in a primary human melanoma. **A** Diagram of gene editing of a human patient melanoma (AV17) from passage in NSG mice. After editing, NSG mice were injected s.c. with WT and IRF2KO tumors, and once the tumors were palpable, they were harvested and analyzed for: **B** the expression of MHC I and PDL1 molecules on the tumors was analyzed by flow cytometer (**C**) mRNA expression of the MHC I pathway components was analyzed by qPCR. Each dot represents a biological replicate. Statistical analysis was calculated by GraphPad Prism, **P < 0.01, ***P < 0.001

We examined a human melanoma patient-derived xenograft (Fig. 2A & B). These primary cells expressed detectable MHC I molecules by immunofluorescence and flow cytometry. These cells were transduced with a lentiviral vector containing Cas9 without (EV) or with IRF2-targeting guides to create IRF2-sufficient and IRF2-deficienct cells that were otherwise isogenic. Cells were then injected in highly immunodeficient NOD scid gamma (NSG) mice to expand these cells. Tumors were collected once they were palpable, enzymatically digested to create single cell suspension and then analyzed for surface MHC I molecules by immunofluorescence and flow cytometry. As show in Fig. 2B, loss of IRF2 dropped MHC I and PDL1 levels substantially (Supp. Fig. 4A). When the expression of MHC I pathway genes was analyzed by qPCR, we found reductions in transcripts of TAP1, TAP2, and ERAP1 genes (other MHC I pathway genes were not examined), which was consistent with our earlier studies with other cancers (Fig. 2C) [6]. The effect of loss of IRF2 dropped the mRNA expression levels of Cas7 and GSDM as well.

**Fig. 3:**
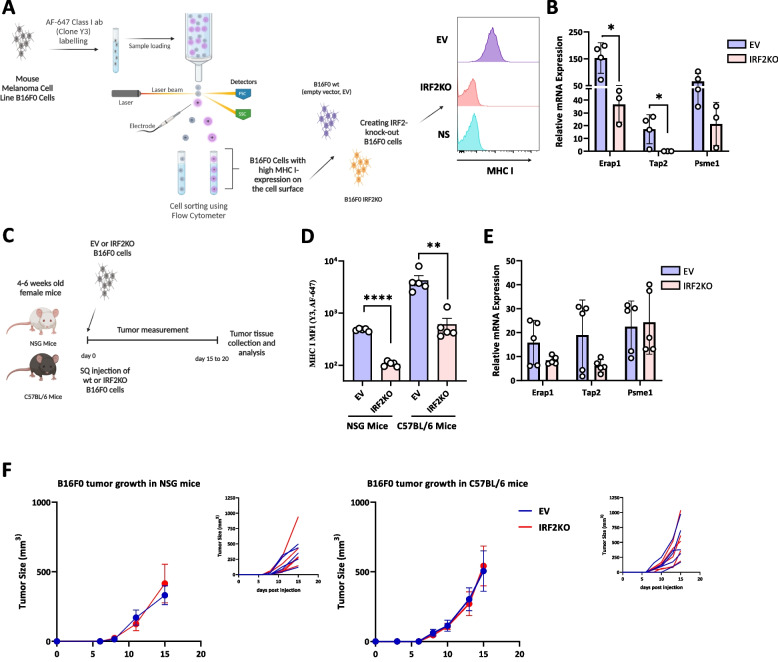
Loss of IRF2 in the mouse melanoma cell line B16F0 reduces the expression of MHC I pathway components but has no effect on B16F0 tumor growth kinetics in NSG or C57BL/6 mice. **A** Diagram of the experimental setup and **B** *In vitro* mRNA expression levels of MHC I pathway components in B16F0 WT (*n=*4) vs IRF2KO (*n=*3) were analyzed by qPCR. **C** Diagram of experiments testing the *in vivo* growth of WT vs IRF2KO B16F0 cells in NSG mice (*n=*10) and C57BL/6 mice (*n=*10) **D** & **E** Tumors from C were collected on day 15 and MHC I expression was analyzed by flow cytometry (**D**) and mRNA expression of the MHC I pathway components was analyzed by qPCR (**E**). Each dot represents a biological replicate and the curves on F show mean +SD. Statistical analysis was calculated by GraphPad Prism, **P < 0.01, ***P < 0.001

To generalize these results, and to do so in a murine system whose immunobiology could be explored in vivo, we performed similar experiments in the B16F0 mouse melanoma cell line. B16FO cells were from a melanoma that arose spontaneously in C57BL6 mice and from which variants arise [15, 16]. For example, the frequently used B16F10 derivative was obtained through 10 serial passages in immunocompetent mice, and during this process lost MHC I expression [17], presumably due to immune-selection. For our studies of the MHC I pathway, we sorted B16F0 cells for uniform high levels of cell surface MHC I molecules (Fig. 3A). These cells were then transduced with a lentiviral vector containing Cas9 without (EV) or with mouse IRF2-targeting guides to create isogenic IRF2-sufficient and IRF2-deficienct cells (Fig. 3A). Similar to primary melanoma cells, loss of IRF2 dropped the surface expression of MHC I and significantly reduced transcripts of TAP2, ERAP1, and PSME1 detected in B16F0 mouse melanoma cells (Fig. 3B).

### The effect of IRF2 on B16 melanoma growth in immunocompetent vs immunodeficient mice

When the isogenic pair of B16 melanomas was injected into highly immunodeficient NSG mice and immunocompetent WT mice, they both grew and did so with the same kinetics (Fig. 3C and F and Supp. Fig.2A and 2B). To determine whether the phenotype of the transplanted tumors changed in vivo, we harvested these cells and analyzed them by flow cytometry and qPCR. After growth in the NSG and WT mice, IRF2-deficient cells still had significantly lower levels of MHC I than the IRF2-sufficient tumors (Fig. 3D, Supp. Fig.4B). Interestingly, when analyzed by qPCR, the expression of the MHC I pathway components, while decreased, were not reduced as much as in the original cells (Fig. 3E).

To summarize, IRF2 loss doesn’t affect growth of the mice melanoma cells B16F0. Finding that WT and IRF2-deficient B16 cells grew rapidly in immunocompetent mice set the stage for the next experiments.

### Effect of IRF2 loss on response to CPI therapy

**Fig. 4:**
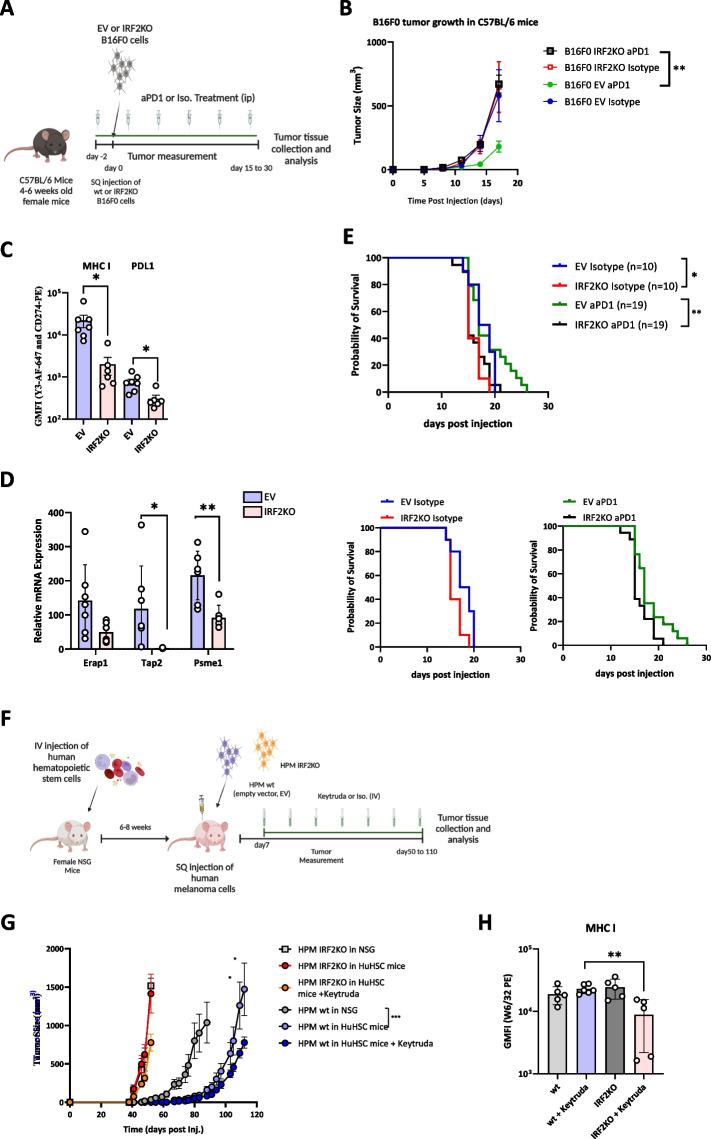
IRF2-deficient human and mouse melanomas are resistant to CPI therapy. **A** Diagram of experiments testing the WT (*n=*14) vs IRF2KO (*n=*12) B16F0 *in vivo* tumor growth in C57BL/6 mice after isotype control or αPD1 treatment. **B** Tumor growth was recorded until the end of the experiment. **C** & **D** Tumors were collected on day 17 and MHC I expression was analyzed by flow cytometry (**C**) and mRNA expression of MHC I pathway components were analyzed using qPCR method (**D**). **E** Another group of C57BL/6 mice (*n=*58) were subcutaneously injected with either WT (*n=*29) or IRF2KO (*n=*29) B16F0 cells and tumor growth was recorded for survival analysis. **F** Diagram of experiments testing the WT (*n=*14) vs IRF2KO (*n=*15) the A17 patient-derived human melanoma growth in NSG (*n=*6) and NSG with HuHSC (*n=*23) mice after isotype control or αPD1 treatment. **G** Tumor growth was recorded until the end of the experiment and (**C**) MHC I expression was analyzed by flow cytometry on day 55 and day 112. **C**, **D** & **H** Each dot represents a biological replicate and the curves on B and G show mean+SD. Statistical analysis was calculated by GraphPad Prism, **P < 0.05, **P < 0.01, ***P < 0.001

Although B16 cells grow aggressively in WT mice, their growth can be slowed in mice treated with CPIs, such as anti-PD1 [18]. Therefore, the immune system still has the potential to detect and slow the growth of this cancer. This situation is similar to what occurs in many melanoma patients that are treated with CPIs. This allowed us to investigate whether loss of IRF2 would lead to resistance to CPI therapy (Fig. 4A).

Injection of anti-PD1 antibody into mice transplanted with IRF2-sufficient B16, significantly slowed the growth of this tumor and extended survival significantly (Fig. 4B, Supp. Figure2B-2F). In contrast, IRF2-deficient B16 were resistant to anti-PD1 therapy and tumor growth and survival were unaffected relative to untreated IRF2-sufficient tumors (Fig. 4B and E). We again analyzed the harvested cells by flow cytometry and qPCR methods and found that IRF2-deficient cells still had significantly lower cell surface levels of MHC I and PDL1 molecules compared to the IRF2-sufficient tumors (Fig. 4C, Supp. Fig.4C). Similarly, the IRF2-null cells had reduced expression of mRNA for MHC I pathway components, e.g. TAP2 and PSME1 (Fig. 4D). Together these results indicate that loss of IRF2 allows the B16 cells to evade the host immune response leading to treatment failure.

### Effect of IRF2 loss on growth and response to CPI therapy in a primary human melanoma

When the isogenic pair of human IRF2-positive and negative primary melanomas were injected into humanized NSG mice, the IRF2-deficient tumors grew more rapidly than their IRF2-positive counterparts (Fig. 4G). This same pattern was observed in NSG mice, indicating that this growth differential was not due to evasion from human anti-tumor adaptive immune responses. Presumably, the loss of IRF2 confers some growth advantage to this melanoma, which is different from what we observed in the mouse melanoma model.

The growth of the WT tumor was significantly slower in humanized NSG mice as compared to NSG animals (Fig. 4G). Therefore, the presence of the human hematopoietic system restrained the WT tumor growth. In contrast, the IRF2-deficient cells evaded this control and grew at the same rapid rate in both NSG and humanized NSG mice (Fig. 4G).

We next investigated how the presence versus absence of IRF2 influenced responses to CPI with anti-PD1. Injection of anti-PD1 antibody into mice transplanted with the IRF2-sufficient human primary melanoma significantly (p ≤0.5) slowed the growth of this tumor (by 1 week). In contrast, IRF2-deficient primary melanomas were resistant to anti-PD1 therapy and tumor growth and survival were unaffected relative to untreated IRF2-sufficient tumors (Fig. 4G). We again analyzed MHC I levels on the harvested tumor cells by flow cytometry. IRF2-deficient cells still had significantly lower cell surface levels of MHC I than the IRF2-sufficient tumors after ICI treatment (Fig. 4H). The MHC I levels in the IRF2-deficient group without ICI were not decreased (Fig. 4H), for reasons that were not clear and likely an outlier as the levels remained low in two other independent experiments (Supp. Fig.2A). In any case, the CPI results indicate that similar to mouse B16 cells, loss of IRF2 allows human primary melanomas to evade the host immune response leading to treatment failure.

### Reversing the immune evasion and resistance to therapy from IRF2-loss with IFN/IFN-inducers

**Fig. 5:**
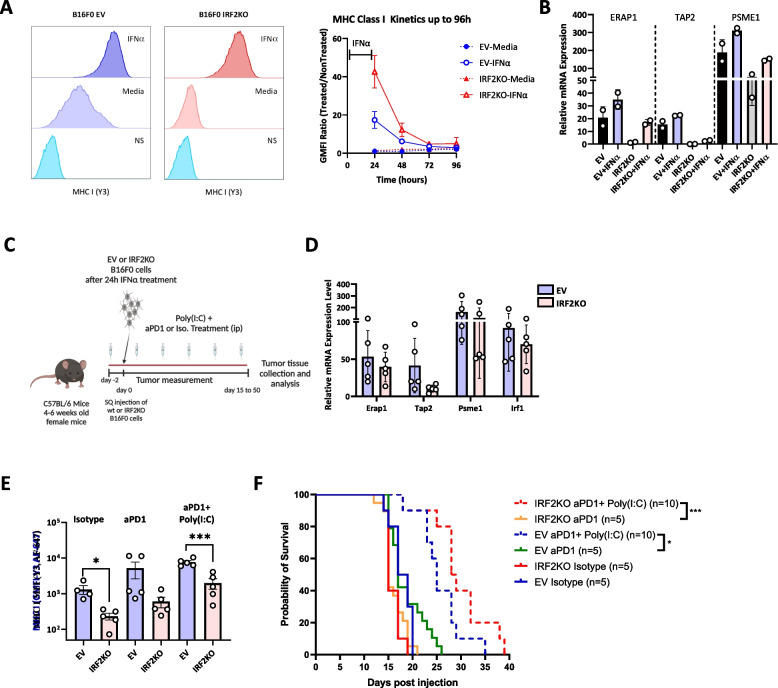
Effect of IFNα on WT and IRF2 KO B16 melanomas. **A** MHC I levels on IRF2KO (*n=*3) and WT cells (*n=*3) after 24 hour stimulation with or without IFNα (histograms) and after withdrawal of IFN (line graph). **B** mRNA expression of MHC I pathway components in WT (*n=*2) and IRF2KO cells (*n=*2) after the IFN treatments. **C** Diagram of the experiment testing the effects of IFNα+poly(I:C) treatment ± anti-PD1 on WT vs IRF2KO B16 melanoma. **D** & **E** Day 15 post tumor injection, tumors were harvested and (**D**) mRNA expression of the MHC I pathway components was analyzed by qPCR and **E** the expression of MHC I molecules on the tumors was analyzed by flow cytometer. Each dot represents a biological replicate and the curves on A. and C. show mean+SD. **F** Another group of C57BL/6 mice (*n=*40) were injected with WT (*n=*20) and IRF2KO (*n=*20) tumors for survival and tumor growth was recorded for survival analysis. Statistical analysis was calculated by GraphPad Prism, **P < 0.01, ***P < 0.001

We have previously shown that in IRF2null dendritic cells, the immune evasion phenotype from IRF2-loss could be reversed by treating cells with IFNγ or IFNα [6]. This led us to investigate whether treatment with IFNs would be able to reverse the immune evasion phenotype and CPI resistance with IRF2-deficient B16 cells. To test this hypothesis, we treated the B16 EV vs IRF2 KO cells with IFNγ or IFNα *in vitro* for 24 hours and then analyzed their phenotype (Fig. 5A, Supp. Fig.3). When analyzed by FACS and qPCR, cell surface MHC I levels (Fig. 5A) and the expression of MHC I pathway component transcripts (Fig. 5B) increased in both WT and IRF2KO cells after the IFN treatments. With IRF2KO cells, IFNs increased MHC I levels above those found in the unstimulated control cells but somewhat below the levels in IFN-stimulated control cells (Fig. 5A and B, Supp. Fig.3).

Given that IFNs could restore MHC I expression, we next investigated whether IFN would reverse the immune evasion phenotype and resistance to treatment with CPI in vivo (Fig. 5C). For this purpose, we injected control EV vs IRF2 KO B16 cells into mice that were treated ± the type I IFN-inducer poly(I:C) and ± anti-PD1. After harvesting the cells, qPCR analysis revealed that the expression of the MHC I pathway component transcripts increased in IRF2 KO tumors (Fig. 5D). We also analyzed the expression of MHC I molecules on the IRF2 KO tumors in the anti-PD1 + poly(I:C)-treated mice, and similarly found that the treatment had increased MHC I levels above those in control isotype-treated and aPD1-treated mice, although these were not quite as high as in the EV B16 in the treated mice (Fig. 5E, Supp. Fig.2). Importantly, the combination of anti-PD1 and poly(I:C) let to a significant prolongation in survival of mice bearing either the EV or IRF2KO melanomas (Fig. 5F). The therapeutic response seen with the IRF2KO tumor was even a bit better than with EV melanoma. Therefore, the treatment with the IFN-inducer completely reversed the resistance of the IRF2null cancer to CPI.

### Role of IRF1 in reversing the immune evasion phenotype in IRF2-deficient B16 melanoma cells

**Fig. 6:**
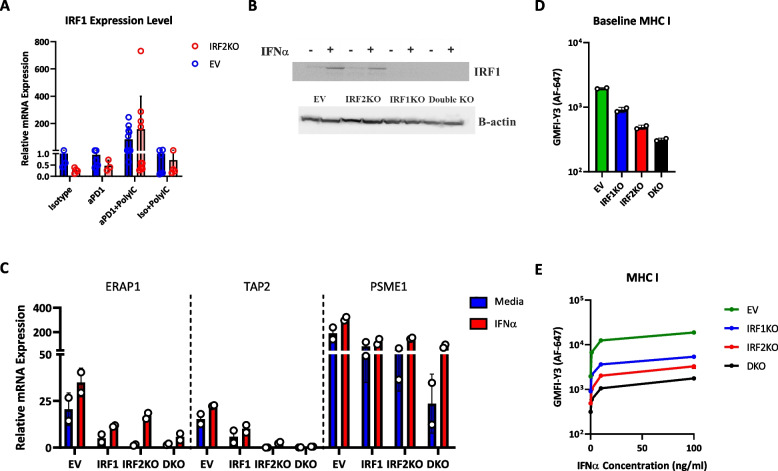
Transcription factor IRF1 substitutes for the loss of IRF2. **A** Same experimental design as 5C and 5D except, IRF1 mRNA expression was analyzed by qPCR. **B-E** EV, IRF1KO, IRF2KO and DKO (IRF1+IRF2KO) B16F0 cells were stimulated with IFNα *in vitro* and analyzed by: **B** Western blot for IRF1 or ß-actin; **C**. qPCR expression for MHC I pathway components (*n=*2/group). **D** & **E** Surface MHC I levels after 0 or 100 ng/ml IFNα treatment for 24 h (*n=*2/group)

The IRF1 and IRF2 transcription factors bind to the same promoter regulatory elements in MHC I pathway genes. IRF2 is constitutively expressed, and IFNs (both type I&II) can modestly increase IRF2 expression, typically by 2-4 fold after 24h treatment [19]. In contrast, IRF1 is often minimally expressed under basal conditions but is markedly induced (often by 4-60-fold) by IFN stimulation [19]. Consistent with this pattern, low levels of IRF1 are detected in B16 melanoma cells by western blot, but IRF1 expression is substantially increased in cells stimulated with IFNα (Fig. 6B, Supp. Fig.5A). To test whether this induction of IRF1 played a role in the IFN-induced reversal of the immune evasion phenotype in IRF2 null B16, we generated and examined IRF2+IRF1 double KO B16 cells (Fig. 6B). The IFN-induced increase in the expression of some MHC I pathway component transcripts that had been seen in IRF2 KO cells *in vitro* was attenuated in IRF2+IRF1 double KO cell (Fig. 6C). Similarly, the IFN-induced increase in surface MHC I levels in the IRF2KO cells was also attenuated when these cells additionally lacked IRF1 (Fig. 6D&E). These results indicated that IRF1 was participating in the restoration of MHC I expression.

**Fig. 7:**
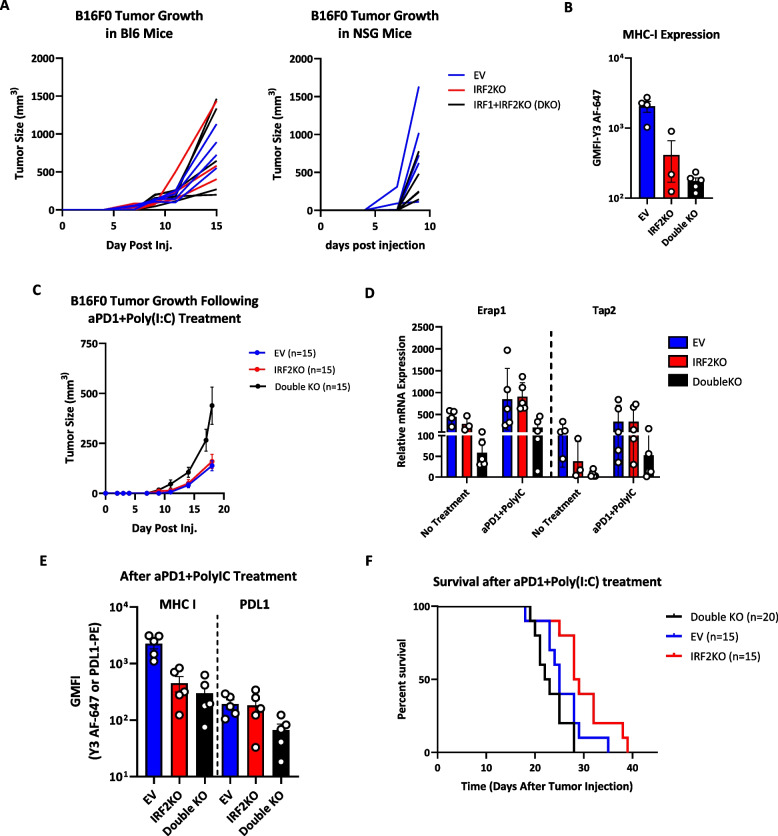
B16 melanoma IRF1+IRF2 double KO cells show impaired responses to IFN inducer Poly(I:C) plus CPI. **A** NSG mice (*n=*10) and C57BL/6 mice (*n=*12) were subcutaneously injected with WT, IRF2KO and IRF1+IRF1KO cells and data display tumor growth in individual mice. **B** Tumors were collected on day 15 and the surface MHC I expression of C57BL/6 tumors was analyzed by flow cytometry. **C** C57BL/6 mice (*n=*45), that were treated with poly(I:C) and aPD1 were injected with IFNα treated (10ng/mL, 24h) control WT vs IRF2KO IRF2+IRF1 (double) KO B16 cells and tumor growth was followed. **D** & **E** On day 17 post tumor injection, *n=*5 mice/group were sacrificed for: **D** mRNA expression analysis of MHC I pathway components using qPCR method and **E** the tumor cell surface expression analysis of MHC I molecules by flow cytometer. Each dot represents the average measurement of the individual tumors collected from mice. **F** Survival analysis of WT or IRF2KO or IRF1+IRF2 KO tumors in C57BL/6 mice. This experiment was repeated twice. Tumor growth curve shows mean+SD (**C**)

To further evaluate the role of IRF1 *in vivo*, we examined the therapeutic effect of anti-PD1+poly(I:C) treatment on IRF1+IRF2 double knockout melanoma cells (Fig. 7A-D). While a therapeutic effect of anti-PD1+poly(I:C) was still seen with these tumors, they were still significantly more resistant to CPI compared to the IRF2 KO cancer cells (Fig. 7E and F). Together, these results show that after being induced by IFN, IRF1 can partially substitute for the function of IRF2 in driving expression of the MHC I pathway.

## Discussion

Our major findings are that IRF2 expression was reduced in subsets of almost all human cancer types and that this reduction had functional consequences. When IRF2 expression was reduced, there was a corresponding reduction in the expression of downstream IRF2 target gene transcripts. This positive correlation was observed between IRF2 expression and that of almost all genes in the MHC I pathway, other IRF2-regulated genes, but not in several housekeeping genes that were examined. Remarkably these correlations were observed in almost all cancer types in the TCGA database. One of these cancer types, melanoma, was selected for further analysis. When IRF2 was knocked out of mouse or primary patient melanomas, there was also a reduction in the expression of IRF2 target genes, establishing a cause-and-effect relationship between IRF2 expression and these genes. In both human and mouse melanomas, loss of IRF2 led to resistance to CPI immunotherapy in preclinical models. Importantly, the IRF2 immune evasion phenotype could be reversed by treatment of cells with type I and II IFN. Moreover, the resistance of IRF2-deficient melanomas to immunotherapy could be restored by treatment with a type I IFN inducer in combination with CPI. In melanomas that were deficient in both IRF1+IRF2, IFN treatment failed to restore the MHC I pathway and reverse the resistance to CPI, indicating that the beneficial effects of the IFN treatment were mediated through the substitutive activity of the transcription factor IRF1. These results elucidate a mechanism that underlies cancer immune evasion through loss of IRF2 expression, which is reversible with currently available biologics, and is likely applicable to many cancers.

The mouse and human melanomas we examined were both ones that were responsive to CPI, but these responses were partial, i.e., CPI treatment slowed their growth and survival was extended, but this did not result in tumor elimination. This is similar to what has been seen in previous studies with these same cancer cells in mice [11, 20–23]. We believe that these are appropriate preclinical models as such partial responses are what is observed in many CPI-treated cancer patients, including ones with melanomas [24–26]. For tumors like B16 melanoma, this may reflect the fact that they are quite aggressive and likely developed some ability to evade immune responses. Against this baseline, loss of IRF2 clearly converted these cancers to being non-responders to CPI. Since our transcriptomic analyses showed that IRF2 levels were “rate limiting” for expression of MHC I pathway components and caspase 7 in most patient cancers, our treatment results predict that patients whose cancers have reduced levels of IRF2 transcripts, which we showed can be frequent, will similarly become more resistant to CPI. This prediction needs to be tested in clinical studies.

Loss of IRF2 may confer resistance to CPI is several ways that are not mutually exclusive. One way is through down regulation of MHC I pathway components. IRF2 positively regulates transcripts for almost all components in the MHC I pathway [6, 10] . Our transcriptomic analyses here suggest that this is the case in subsets of many human cancer types. Since as discussed above, IRF2 levels seem to be limiting, the reduction in IRF2 expression will affect the expression of multiple components of the pathway regulating MHC I expression with the net effect in the impairment of the MHC I antigen pathway expected to be additive. While we didn’t resolve which of the IRF2-regulated MHC I components became functionally limiting in these cancer cells, we had previously shown that in IRF2 null cells, TAP and ERAP1 functions (peptide transport into the ER and subsequent peptide trimming) are inhibited, as is overall antigen presentation [6]. In any case, the important point is that the net effect of the reduction of MHC I pathway transcripts is a decrease in the number of peptide-MHC I complexes on the cell surface. This should make it harder for CD8 T cells to recognize the IRF2^low^ cancers. Where examined, low MHC I levels in cancers have been associated with poorer responses to CPI [5].

Another way that loss of IRF2 might confer resistance to CPI is through a reduction in caspase 7. This is because as discussed above, reductions in caspase 7 could impair the ability of CD8 T cells to kill cancer cells with granulysin. However, this would not cause resistance in our mouse melanoma because murine CD8 T cells lack granulysin. Yet another way that IRF2 could lead to immune evasion is by decreasing the repression of PDL-1. However, while this can occur in some mouse and human cells [6, 10], we did not observe this effect in the present study. Thus, IRF2 repression of PDL-1 may be cell line specific. Finally, it is possible that a reduction in other IRF2-dependent processes (e.g., gasdermin D), may contribute to the immune evasion phenotype and resistance to CPI.

NK cells can also kill tumor cells. But, they recognize cells that do not express receptors MHC I and recognition of surface MHC I molecules inhibits NK cytotoxicity. Consequently, some cells lacking MHC I molecules are killed by NK cells [27]. In a preliminary experiment we found that the growth rate of IRF2-sufficient versus IRF2-null B16 cells was identical in mice that were depleted of NK cells (Supp. Fig.5B). Perhaps this is because the IRF2-deficient tumor cells are able to evade NK cells because they still expresses some MHC I molecules and/or have decreased caspase 7, however further studies are needed to determine whether and to what extent NK cells can recognize and control IRF2-deficient cancers. Finally some additional as yet unknown targets of IRF2 in CD8 or NK cell may be involved.

IRF-2 is a transcription factor with many gene targets and thereby plays diverse roles. IRF-2 has been implicated in the regulation of cell growth and differentiation in various cell types influencing the expression of genes involved in cell cycle regulation, apoptosis, and differentiation processes [28]. We couldn’t detect any proliferation and growth differences in IRF2-deficient mouse melanoma cell line either *in vivo* or *in vitro*. On the other hand, the IRF2-deficient human patient melanoma grew more aggressively in both severely immunodeficient NSG mice and human hematopoietic stem cell transplanted, immunocompetent NSG mice. These observations may be related to earlier studies showing oncogenic effects of IRF2 suppression [29], although overexpression of IRF2 has also been reported to cause oncogenic transformation in pancreatic cancers and leukemias [30–32]. Perhaps how IRF2 expression affects tumor growth depends on the particular cancer. Downregulation of IRF2 in human melanomas might be creating a double whammy via downregulation of MHC I antigen presentation to escape immune detection and releasing the brakes on cell proliferation at the same time. It will be of interest in future studies to examine whether this is the case in other tumors and experimental settings. Whether the IRF2 effects on the human melanoma growth contribute to CPI resistance to in our system is not resolved by our data, although other rapidly growing tumors like B16 do respond to CPI.

IRF2 is constitutively expressed in cells and our data showed that this expression is important for maintaining the activity of the MHC I pathway and expression of MHC I molecules. IRF2’s close relative, IRF1, is induced by type I and II IFN-stimulation and binds competitively to the same DNA motifs as IRF2. IRF1 and IRF2 are both activators of the MHC I pathway genes. Importantly we found that type I and II IFN stimulation of IRF2-null melanomas restored their MHC I pathway and MHC I molecule expression and that this was in part dependent on IRF1. Similarly, systemic treatment with a type I IFN inducer, poly I:C, reversed resistance of IRF2 null cells to CPI *in vivo* and this salutatory effect was also dependent on IRF1 in the cancer cells. This demonstrates that IFN induction of IRF1 can reverse the immune evasion consequences of the loss of IRF2. When both IRF1 and IRF2 were absent, type I IFN treatment could not restore MHC I expression or reverse resistance to CPI, confirming the concept that type I IFN can induce IRF1 and restore immunogenicity of the tumor cells and host cytotoxic response to them. These findings have some likely translational implications. Treatment of melanoma patients with pegylated IFNa2b as an adjuvant therapy post-surgical resection was approved by the FDA [33]. Our results suggest a potential mechanism that might contribute to this agent’s efficacy in treating melanoma. Our results further suggest that in patients with IRF2^low^ melanomas or potentially other IRF2^low^ cancers, adding IFN treatment to CPI therapy might improve efficacy. In fact, a recent study found that prior treatment with pegylated-IFN-alfa-2b increased the effectiveness of adjuvant pembrolizumab (anti-PD-1 treatment) in patients with surgically removable advanced melanoma, although IRF1 and IRF2 status was not evaluated [34]. Since type II interferon can also induce IRF1, and this cytokine was FDA-approved for other indications, future studies should examine the effects of type II IFN on melanomas that are IRF2-deficient and its potential as an adjuvant therapy with CPI.

Our studies suggest low levels of IRF2 may be responsible for some of the poor immunogenicity of many tumors and that in preclinical studies, type I or II interferons can induce tumor expression of IRF2, enhancing MHC I expression and lead to better rejection, including in combination with CPI treatment. Given our findings, in future studies it will also be of interest to examine whether IRF2 expression levels in tumor biopsies might be a biomarker for subsequent responsiveness to CPI either by itself or with other markers. Similarly, it would be on interest to determine whether a cancer’s levels of IRF1 and IRF2 might be biomarkers for tumors that might benefit IFN treatment to improve the efficacy of CPI.

## Supplementary Information

Supplementary Material 1. 

Supplementary Material 2.  Supp. Fig 1. Differential expression of IRF2 gene in tumor and corresponding normal tissue from patients with the indicated cancer types (TCGA abbreviations).

Supplementary Material 3. Supp. Fig 2. (A) A17  patient-derived human melanoma. was implanted into  NSG (*n*=9) and NSG with HuHSC (*n*=15) mice. On day 35 (IRF2KO) and day 77 (EV) tumors were harvested and MHC I expression was analyzed by flow cytometry. (B-E) B16F0 EV or IRF2 KO cells were implanted into humanized C57Bl/6 mice and treated as indicated. Cell surface MHC I (C) and PDL1 (D) expression levels and tumor weight (E) of B16F0 EV vs IRF2KO tumors were quantified on day 15-17 in vivo. (F) shows corresponding tumor growth data.

Supplementary Material 4. Supp. Fig 3. (A) Effect of IFNα and IFNγ on WT and IRF2KO B16 melanomas. (B) MHC I levels on IRF2KO (*n*=3) and WT cells (*n*=3) after 24 hour stimulation with 10 ng/ml IFNα or IFNγ. (C) mRNA expression of MHC I pathway components in WT (*n*=2) and IRF2KO cells (*n*=2) after the IFN treatments.

Supplementary Material 5. Supp. Fig 4. (A) Corresponding histograms of Fig.2B showing MHC I and PDL1 levels on IRF2KO (*n*=5) and WT cells (*n*=3) tumors in NSG mice. (B) Corresponding histograms of Fig.3D showing MHC I levels on IRF2KO (*n*=5) and WT cells (*n*=5) tumors in NSG mice. (C) Corresponding histograms of Fig.4C showing PDL-1 levels on IRF2KO (*n*=5) and WT cells (*n*=7) tumors in C57Bl/6 mice after aPD1 or isotype treatment. 

Supplementary Material 6. Supp. Fig 5. (A) IRF1 and IRF2 mRNA expression levels in WT B16 melanoma cells with (*n*=4) or without (*n*=3) 10 ng/ml IFNα treatment for 24h in vitro. (B) In vivo growth of WT (*n*=3) vs IRF2KO (*n*=3) B16F0 cells in C57BL/6 mice that were treated with anti-mouse NK1.1 antibody (clone: PK136, BioXcell) every 3 days starting at day -2.

## Data Availability

All figures and tables, including the supplementary materials, are cited within the manuscript accordingly. Reference links are available for all cited articles.

## Abbreviations

CASP7: Caspase-7
ChIP: Chromatin immunoprecipitation
CPI: Checkpoint inhibitor
ERAP: Endoplasmic reticulum (ER) aminopeptidase
EV: Empty vector
GSMD: Gasdermin D
IFN: Interferon
IRF: Interferon regulatory factor
GMFI: Geometric mean fluorescent intensity
MHC: Major histocompatibility complex
PD1: Programmed cell death protein 1
PDL1: Programmed death-ligand 1 (CD274)
PSMB: Proteasome subunit beta type
PSME1: Proteasome Activator Subunit 1
sgRNA: Single guide RNA (short guide RNA)
TAP: Transporter associated with antigen processing
TAPBP: Tapasin or TAP binding protein
UCB: Umbilical cord blood
WT: Wild-type
