# Supplementary material for: IRF2 loss is associated with reduced MHC I pathway transcripts in subsets of most human cancers and causes resistance to checkpoint immunotherapy in human and mouse melanomas
Source: J Exp Clin Cancer Res. 2024 Oct 2;43:276. doi: 10.1186/s13046-024-03187-5 (PMC11446056; doi:10.1186/s13046-024-03187-5)
Supplement: Supplementary file 1 — Supplementary Material 1. [file 13046_2024_3187_MOESM1_ESM.docx]

**Table-1A:** Transcription factor IRF2 target genes obtained from ChIP-Atlas. IRF2 binding score obtained from MACS2 and STRING analysis and binding scores graded as: **+++** for >750/1000; **++** for >500/100 and <750/1000; **+** for <500/1000; and **0** for <250/1000. **na**: not applicable for the genes that are not reported. (data set ID: CD34+ cells: SRX160847 and K562 cell line: SRX2423855 and SRX2423856)

| Potential IRF2 Binding Score at ±1 kb of Transcription Start Site | | | |
| --- | --- | --- | --- |
| **Analyzed Cell Type** | | **CD34+ Cells K-562 Cell Line** | |
| **MHC I Pathway Genes** | β2M | + | +++ |
|  | ERAP1 | +++ | +++ |
|  | ERAP2 | + | +++ |
|  | PDIA3 | na | na |
|  | PSMB8 | +++ | +++ |
|  | PSMB9 | +++ | +++ |
|  | PSMB10 | +++ | +++ |
|  | PSME1 | +++ | +++ |
|  | TAP1 | +++ | +++ |
|  | TAP2 | +++ | +++ |
|  | TAPBP | +++ | +++ |
|  | TAPBPL | + | +++ |
|  | CD274 | ++ | +++ |
|  | CASP7 | +++ | +++ |

**Table-1B:** Transcription factor IRF1 target genes obtained from ChIP-Atlas. IRF1 binding score obtained from MACS2 and STRING analysis and binding scores graded as: **+++** for >750/1000; **++** for >500/100 and <750/1000; **+** for <500/1000; and **0 (-)** for <250/1000. **na**: not applicable for the genes that are not reported. (Data set ID: SRX212661 and SRX212662).

| Potential IRF1 Binding Score at ±1 kb of Transcription Start Site | | | |
| --- | --- | --- | --- |
| **Analyzed Cell Type** | | **Blood CD14+ Monocytes**  **Cultured with MCSF (10ng/ml) for 24 hours.** | **Blood CD14+ Monocytes**  **Cultured with MCSF (10ng/ml) and IFN-gamma (100U/ml) for 24 hours.** |
| **MHC I Pathway Genes** | β2M | 0 | +++ |
|  | ERAP1 | 0 | +++ |
|  | ERAP2 | 0 | +++ |
|  | PDIA3 | na | na |
|  | PSMB8 | +/- | +++ |
|  | PSMB9 | +/- | +++ |
|  | PSMB10 | 0 | +++ |
|  | PSME1 | +/- | +++ |
|  | TAP1 | +/- | +++ |
|  | TAP2 | +/- | +++ |
|  | TAPBP | 0 | +++ |
|  | TAPBPL | 0 | +++ |
|  | CD274 | 0 | +++ |
|  | CASP7 | 0 | +++ |
