# Supplementary figures and images for: IRF2 loss is associated with reduced MHC I pathway transcripts in subsets of most human cancers and causes resistance to checkpoint immunotherapy in human and mouse melanomas
Source: J Exp Clin Cancer Res. 2024 Oct 2;43:276. doi: 10.1186/s13046-024-03187-5 (PMC11446056; doi:10.1186/s13046-024-03187-5)

## Slide 1
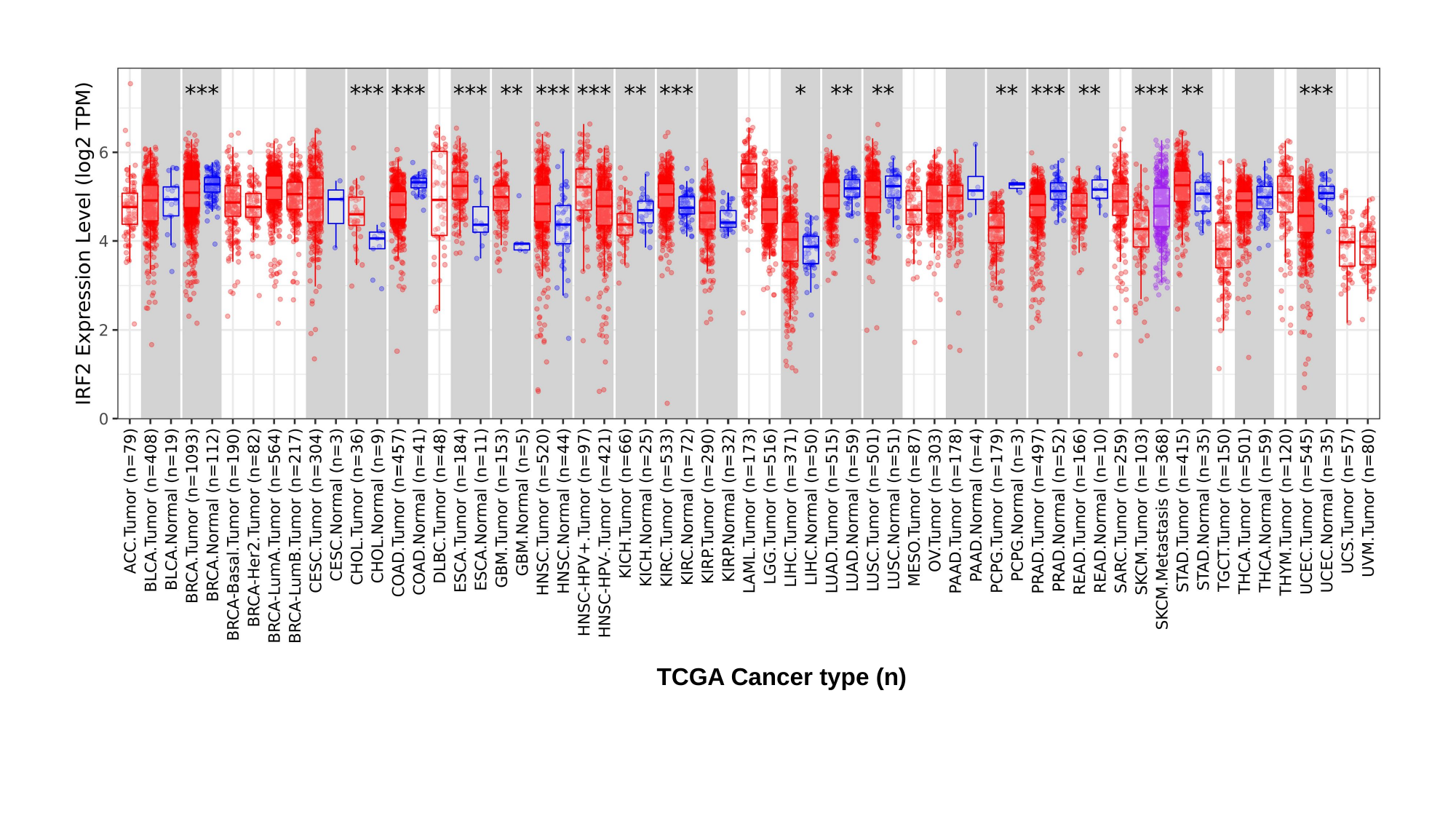

TCGA Cancer type (n)

Supplement: Supplementary file 2 — Supplementary Material 2. Supp. Fig 1. Differential expression of IRF2 gene in tumor and corresponding normal tissue from patients with the indicated cancer types (TCGA abbreviations). [file 13046_2024_3187_MOESM2_ESM.pptx]

## Slide 1
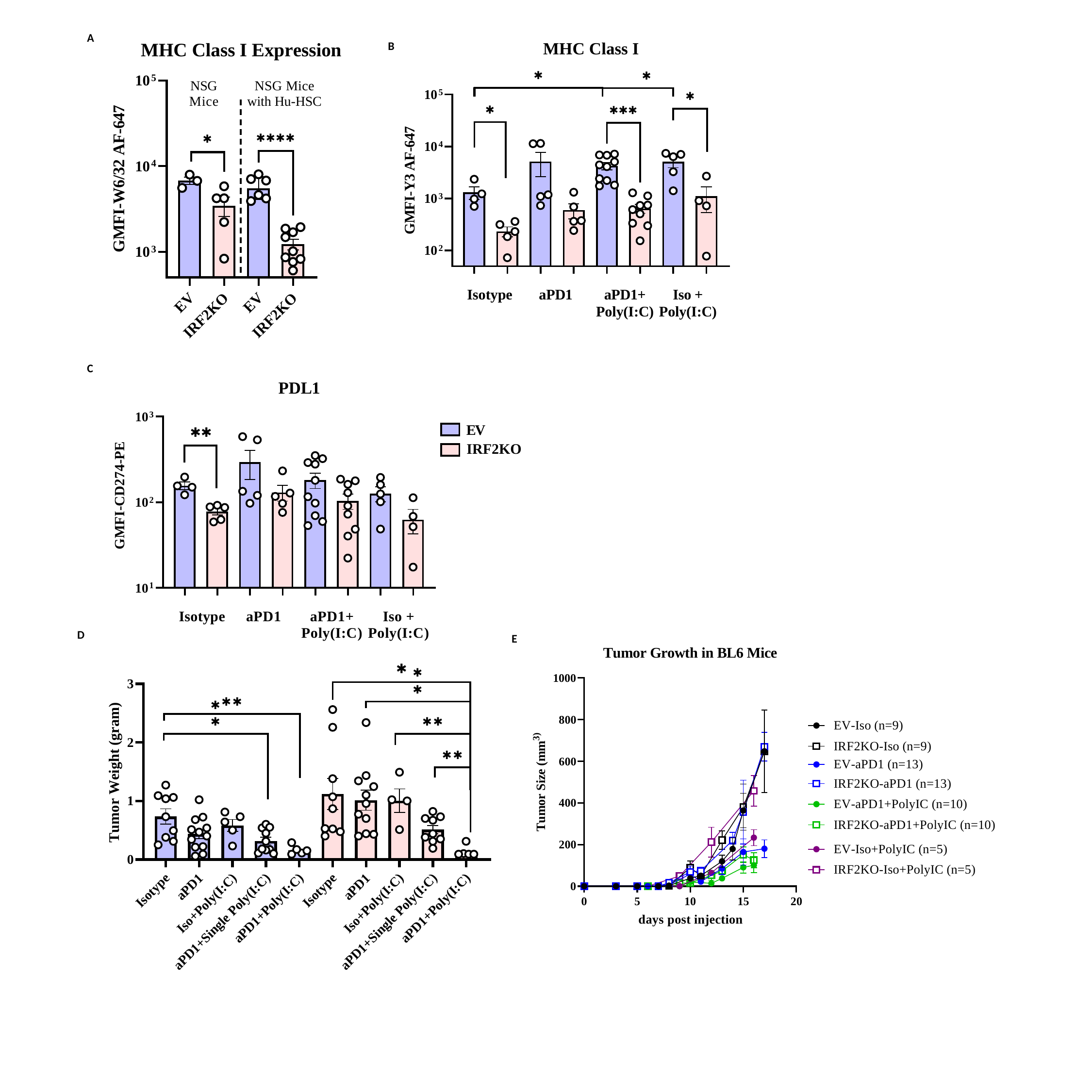

A
B
C
GMFI-CD274-PE
D
E

Supplement: Supplementary file 3 — Supplementary Material 3. Supp. Fig 2. (A) A17 patient-derived human melanoma. was implanted into NSG (n=9) and NSG with HuHSC (n=15) mice. On day 35 (IRF2KO) and day 77 (EV) tumors were harvested and MHC I expression was analyzed by flow cytometry. (B-E) B16F0 EV or IRF2 KO cells were implanted into humanized C57Bl/6 mice and treated as indicated. Cell surface MHC I (C) and PDL1 (D) expression levels and tumor weight (E) of B16F0 EV vs IRF2KO tumors were quantified on day 15-17 in vivo. (F) shows corresponding tumor growth data. [file 13046_2024_3187_MOESM3_ESM.pptx]

## Slide 1
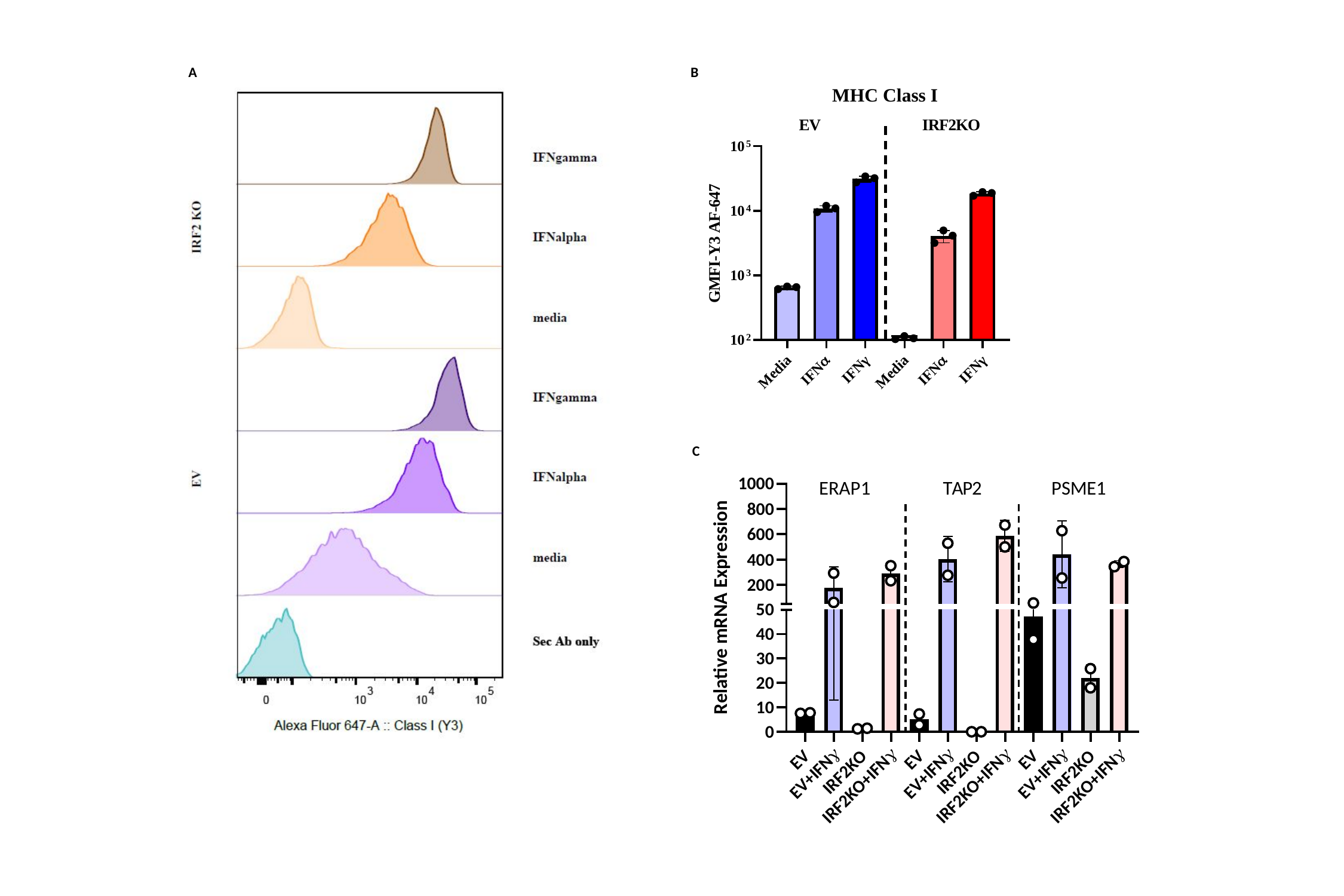

A
B
C

Supplement: Supplementary file 4 — Supplementary Material 4. Supp. Fig 3. (A) Effect of IFNα and IFNγ on WT and IRF2KO B16 melanomas. (B) MHC I levels on IRF2KO (n=3) and WT cells (n=3) after 24 hour stimulation with 10 ng/ml IFNα or IFNγ. (C) mRNA expression of MHC I pathway components in WT (n=2) and IRF2KO cells (n=2) after the IFN treatments. [file 13046_2024_3187_MOESM4_ESM.pptx]

## Slide 1
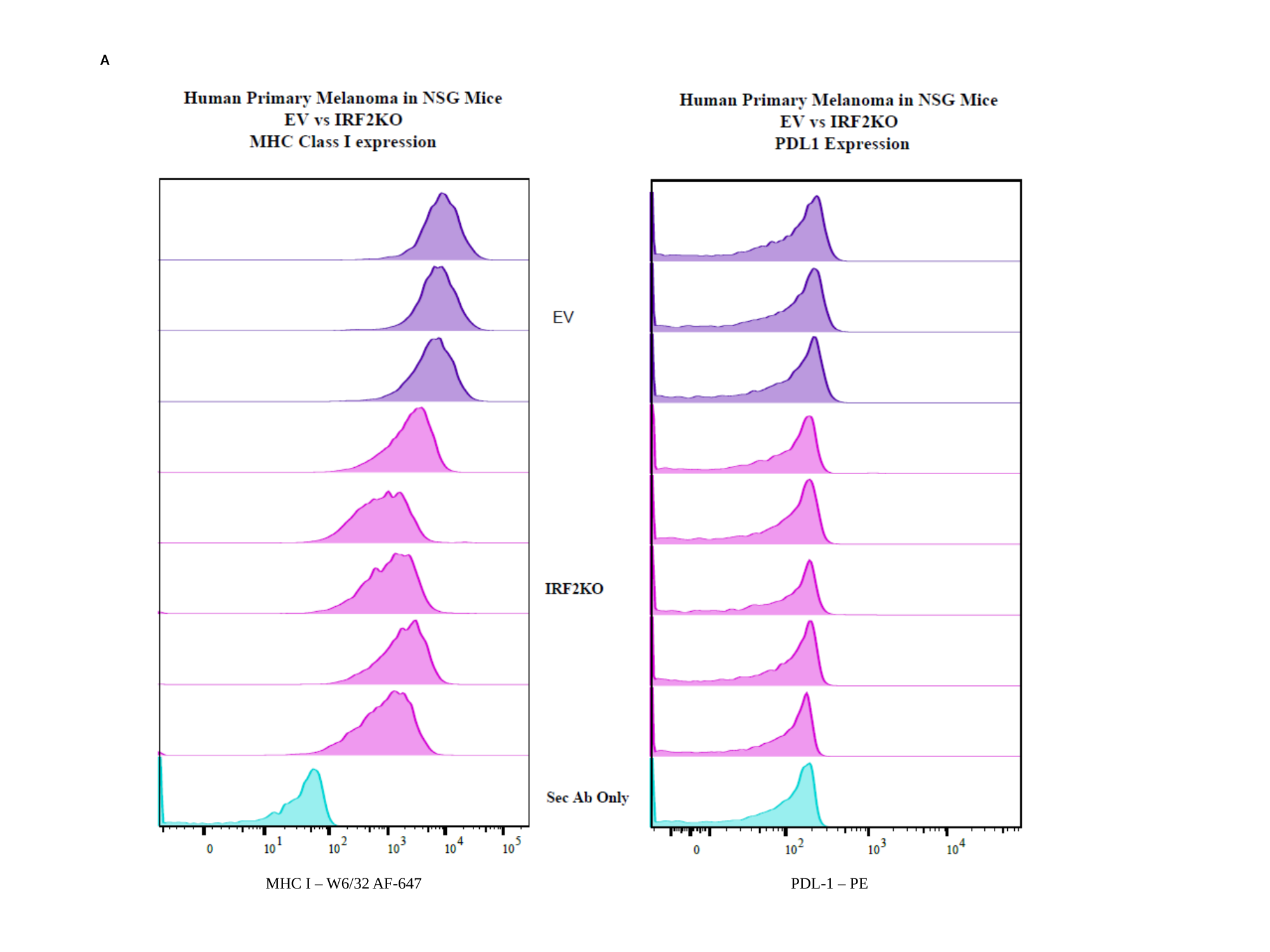

A
MHC I – W6/32 AF-647
PDL-1 – PE

## Slide 2
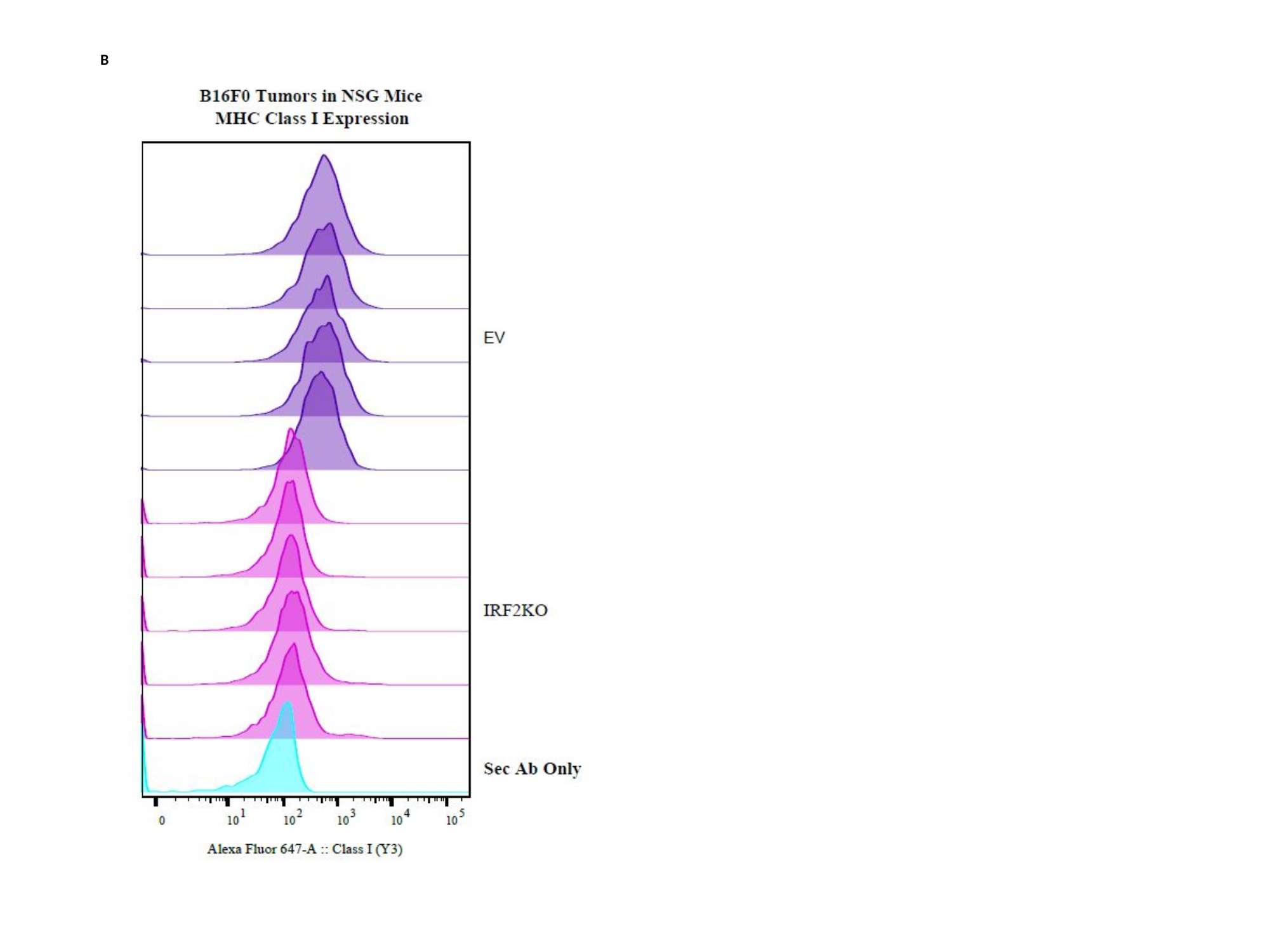

B

## Slide 3
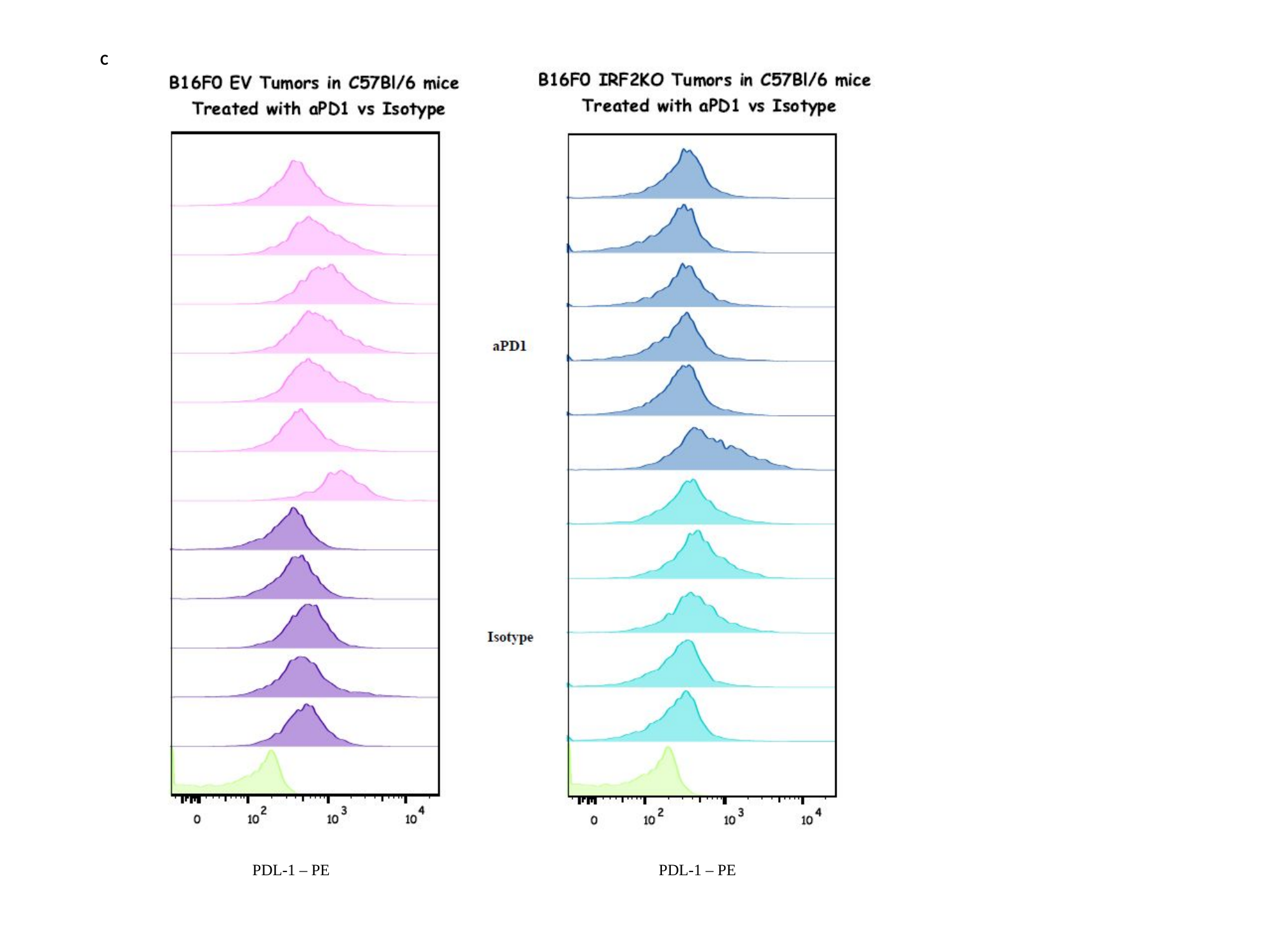

C
PDL-1 – PE
PDL-1 – PE

Supplement: Supplementary file 5 — Supplementary Material 5. Supp. Fig 4. (A) Corresponding histograms of Fig.2B showing MHC I and PDL1 levels on IRF2KO (n=5) and WT cells (n=3) tumors in NSG mice. (B) Corresponding histograms of Fig.3D showing MHC I levels on IRF2KO (n=5) and WT cells (n=5) tumors in NSG mice. (C) Corresponding histograms of Fig.4C showing PDL-1 levels on IRF2KO (n=5) and WT cells (n=7) tumors in C57Bl/6 mice after aPD1 or isotype treatment. [file 13046_2024_3187_MOESM5_ESM.pptx]

## Slide 1
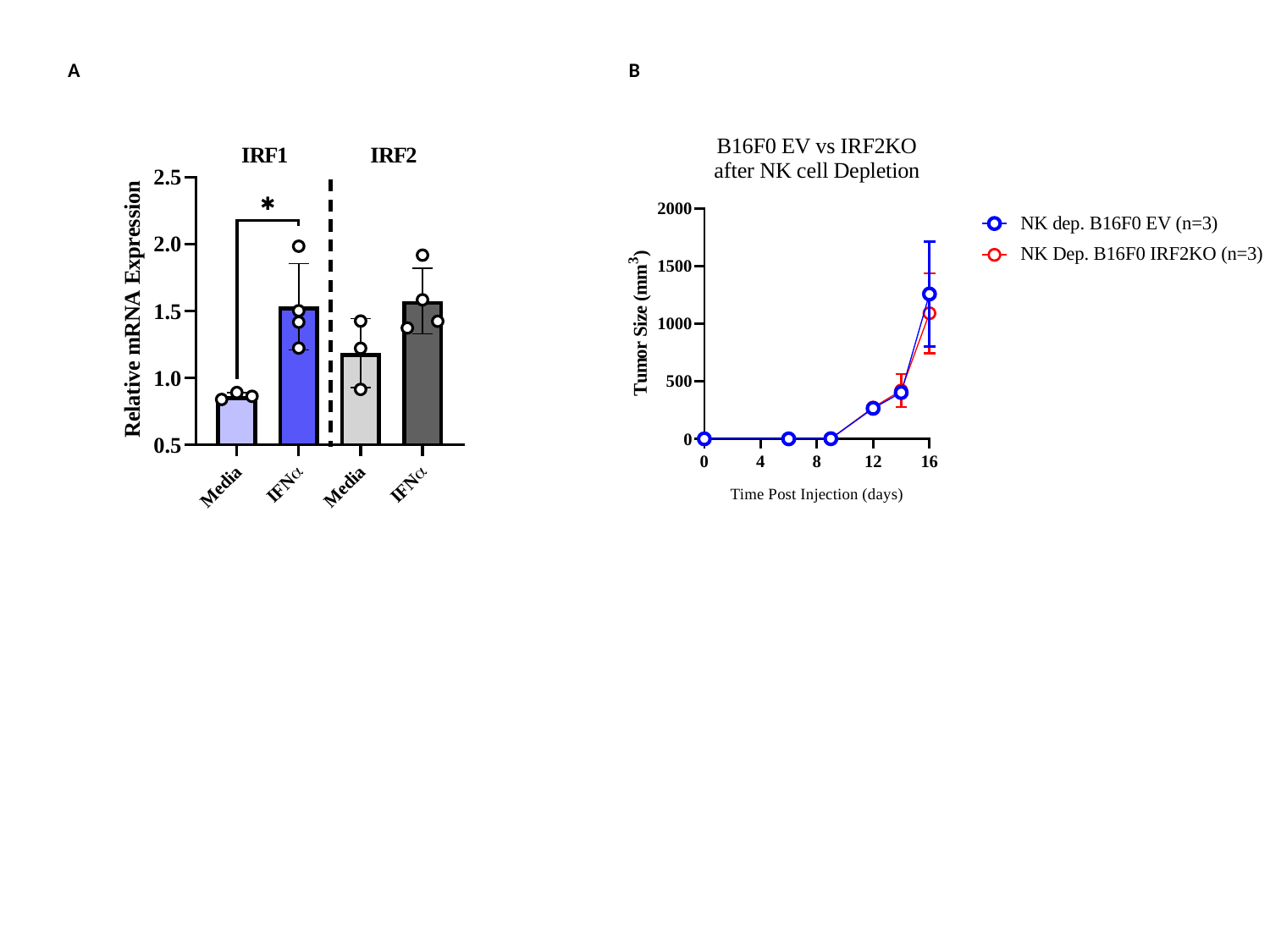

A
B

Supplement: Supplementary file 6 — Supplementary Material 6. Supp. Fig 5. (A) IRF1 and IRF2 mRNA expression levels in WT B16 melanoma cells with (n=4) or without (n=3) 10 ng/ml IFNα treatment for 24h in vitro. (B) In vivo growth of WT (n=3) vs IRF2KO (n=3) B16F0 cells in C57BL/6 mice that were treated with anti-mouse NK1.1 antibody (clone: PK136, BioXcell) every 3 days starting at day -2. [file 13046_2024_3187_MOESM6_ESM.pptx]
